# Novel Botulinum Neurotoxins: Exploring Underneath the Iceberg Tip

**DOI:** 10.3390/toxins10050190

**Published:** 2018-05-10

**Authors:** Domenico Azarnia Tehran, Marco Pirazzini

**Affiliations:** Department of Biomedical Sciences, University of Padova, Via Ugo Bassi 58/B, 35131 Padova, Italy

**Keywords:** botulinum neurotoxins, botulism, serotypes, subtype, neuromuscular junction

## Abstract

Botulinum neurotoxins (BoNTs), the etiological agents of botulism, are the deadliest toxins known to humans. Yet, thanks to their biological and toxicological features, BoNTs have become sophisticated tools to study neuronal physiology and valuable therapeutics for an increasing number of human disorders. BoNTs are produced by multiple bacteria of the genus *Clostridium* and, on the basis of their different immunological properties, were classified as seven distinct types of toxin. BoNT classification remained stagnant for the last 50 years until, via bioinformatics and high-throughput sequencing techniques, dozens of BoNT variants, novel serotypes as well as BoNT-like toxins within non-clostridial species have been discovered. Here, we discuss how the now “booming field” of botulinum neurotoxin may shed light on their evolutionary origin and open exciting avenues for future therapeutic applications.

## 1. Introduction

Botulinum neurotoxins (BoNTs) are the etiological agents of botulism, a rare but severe disease for vertebrates, causing neuroparalysis at peripheral nerve endings [1,2,3]. BoNTs are metalloproteases that cleave members of the “soluble *N*-ethylmaleimide-sensitive-factor attachment receptor” (SNARE) protein family, namely, vesicle-associated membrane protein (VAMP, also known as synaptobrevin), synaptosomal-associated protein 25 (SNAP-25) and syntaxins (mainly syntaxin-1A/1B). SNARE proteins mediate the fusion of synaptic vesicles with the neuronal presynaptic plasma membrane [4,5] and their proteolysis leads to a failure of neurotransmission, thereby causing the deadly flaccid paralysis typical of botulism [2,3,6]. BoNTs are a matter of concern, as they are the deadliest toxins known to humans, with an estimated lethal dose in the low ng/Kg range [1]. At the same time, the neurospecific activity, reversibility and limited diffusion upon local injection make them safe and successful drugs for a steadily increasing number of human disorders characterized by hyperfunction of peripheral nerve terminals [1,7,8,9]. In addition, BoNTs are considered as valuable and sophisticated tools for studying neuron physiopathology [10,11,12]. For all these reasons, BoNTs are considered Janus-faced molecules, being both some of the safest drugs on the market and potential bioweapons at the same time [13].

BoNTs are produced by sporulating and anaerobic Gram-positive bacteria of the genus *Clostridium*, which consists of more than 150 species. These bacteria are ubiquitous in the environment, where they are mainly present as spores that germinate in favorable conditions (anaerobiosis, nutrient availability and low pH) [3]. During vegetative growth, not solely the foremost *Clostridium botulinum*, but additional species, such as *Clostridium butyricum*, *Clostridium baratii* and *Clostridium argentinensis*, become toxigenic and produce the neurotoxins. Using antisera from animals immunized with specific toxin types, BoNTs have been classified into seven different serotypes indicated with alphabetical letters (from BoNT/A to /G) [2,14]. After the discovery of the last serotype (BoNT/G), such a nomenclature remained unchanged for about 50 years. Then, since the introduction of DNA sequencing methodologies, this static view has been continuously evolving and many alternative variants of BoNT serotypes have been emerging. Mosaic toxins, deriving from the hybridization of two serotypes, or toxins related to a parental serotype but displaying divergent amino acid sequences, referred to as subtypes, were identified [15,16]. Presently, faster and cheaper high-throughput sequencing techniques, combined with computational biology, are again changing the scenario [16]. Novel serotypes, many subtypes and mosaic toxins are now more easily detected and the number of BoNTs is constantly and rapidly increasing. Moreover, genomic data-mining is now revealing the unexpected and remarkable finding that BoNT-like genes are also present within non-clostridial species such as *Weissella*, *Enterococcus* and *Chryseobacterium* [17,18].

Here, we review the recent findings about this booming field of botulinum neurotoxins and discuss how these “novel BoNTs” may shed light on their evolutionary origin and inspire researchers to design novel therapeutic tools to expand the present clinical usage.

## 2. BoNTs’ Molecular Architecture and Mechanism of Nerve Endings Intoxication

**Figure 1:**
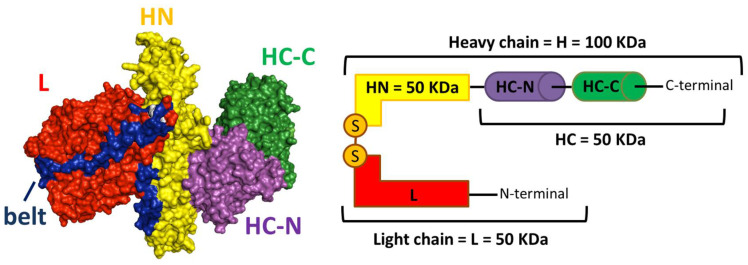
Dichain molecular architecture of botulinum neurotoxins (BoNTs) consisting of four functional subdomains. The crystal structure of BoNT/A (PDB: 3BTA [19]) is shown as a space-filling model (left panel). The functional subdomains are labeled in different colors: HC-C (in green) and HC-N (in purple) mediate toxin binding to the neuronal plasma membrane; HN (in yellow) mediates the translocation of L metalloprotease (in red) into the cytosol. HN and the L chain are kept together via the interchain disulfide bond (in white) and by extensive protein–protein interactions also involving a string of HN residues, known as the belt (in blue), that entirely encircles the catalytic domain. In the right panel, the dichain structure and the functional subdomains are shown as a schematization with the same colors.

The structure and the mechanism of action of BoNTs have been shaped during evolution to exploit essential features of neuron physiology [2,16]. Serotypes have relatively low sequence identity but display a highly preserved molecular architecture arranged in a catalytic light chain of 50 kDa (L) and a heavy chain of 100 KDa (H), held together by a strictly conserved interchain disulfide bond (Figure 1) [19,20]. According to their functions, H and L can be divided into four subdomains: (i) HC-C (25 kDa) mediates the binding and the subsequent internalization/trafficking of the toxin within nerve terminals [21,22]; (ii) HC-N (25 kDa) contributes to the binding by interacting with membrane lipid microdomains [23,24] and by forming, at the interface with HC-C, a crevice where *N*-glycans of glycosylated receptors can be accommodated [25,26]; (iii) HN (50 kDa) mediates the translocation of the catalytic protomer from the luminal part of endocytic compartments into the cytosol [27,28,29]; and (iv) L is a zinc metalloprotease specific for the SNARE proteins [30,31,32], the three essential components governing the neuroexocytosis process (Figure 1) [4,5].

**Figure 2:**
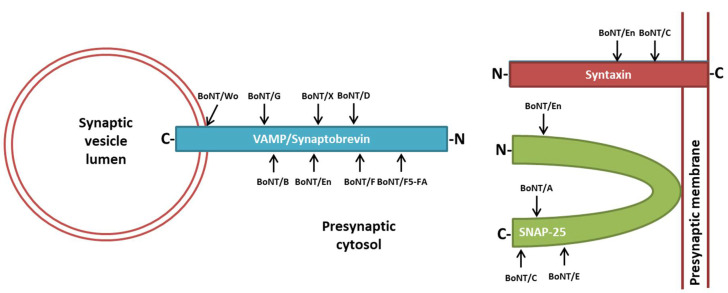
Cleavage sites within SNARE (soluble *N*-ethylmaleimide-sensitive-factor attachment receptor) proteins by the seven BoNT serotypes (from BoNT/A to /G) and by the newly identified BoNT/FA (also known as BoNT/HA or BoNT/H), BoNT/Wo, BoNT/X and BoNT/En (also known as eBoNT/J).

Extensive studies using in vitro and in vivo models pinpointed the five-step mechanism by which BoNTs exert their highly selective and potent neurotoxic activity [33,34,35,36]. Once in the perineuronal fluid compartment, BoNTs bind the unmyelinated presynaptic plasma membrane of skeletal and autonomic nerve endings via two independent receptors [1,21,22,37]. With no known exception, all BoNTs bind polysialogangliosides, a class of glycolipid molecules exclusively expressed on neuronal plasma membranes. Such a binding mediates the accumulation of BoNTs onto the membrane of peripheral nerves and facilitates the interaction with a second receptor, allowing toxin internalization [2,37]. This functional receptor is the luminal domain of a synaptic vesicle protein, which is different among serotypes (Table 1). BoNT/A binds one isoform of the transmembrane protein SV2 (SV2A, B or C) [38,39,40] like BoNT/E, except for SV2C [41]. BoNT/B, BoNT/DC and BoNT/G interact with the luminal domain of synaptotagmin-1/2 [42,43,44]. Recent studies suggest that SV2C glycosylation plays a pivotal role on BoNT/A binding/internalization [25,45]. Considering that protein glycosylation can vary among individuals [46,47,48], this finding may explain why humans display variable susceptibility to the same therapeutic doses of BoNT/A [1]. Even though the importance of glycosylation in protein receptor has been shown only for the BoNT/A and SV2C, synaptotagmin-1/2 also are glycosylated nearby the BoNT binding motif, leaving open the possibility of a role for *N*-glycans in the binding of BoNT/B, BoNT/DC and BoNT/G [26]. Interestingly, BoNT/DC was recently shown to have a ganglioside binding site that, differently from all BoNTs, recognizes a single sialic acid residue rather than extended portions of the polysialoganglioside structure. As a consequence, BoNT/DC has been suggested to bind noncomplex gangliosides and a broad range of binding partners harboring sialic acids on their molecule [49]. This feature of BoNT/DC is surprising, as it is difficult to reconcile its extreme toxicity (LD_50_~0.05 ng/kg [50]) with such a broad and unspecific binding capacity. BoNT/D binding remains controversial, with data indicating multiple bindings to polysialogangliosides and other data showing the need for SV2 proteins [51,52,53]. So far, no protein receptors have been reported for BoNT/C, suggesting that it may entirely depend on the interaction with polysialogangliosides [54,55,56]. Upon binding, BoNT/A is rapidly internalized by synaptic vesicles [57,58,59], whereas the endocytic compartment used by other BoNT serotypes remains to be identified. Thereafter, the L chain has to reach the cytosol. The translocation process requires a concerted rearrangement of the HN domain and of the L chain, which is triggered by the acidification of the endocytic compartment. At low pH, BoNTs form a membrane channel, which is associated with the passage of the L chain [60,61,62]. Despite different mechanisms having been envisaged, the real molecular process is still poorly understood [27,28,29]. However, it is clear that some unfolding of L is necessary and that the interchain disulfide bond must remain intact during the initial phase of membrane translocation. Consequently, the disulfide bond must be reduced, and L has to be properly refolded to be released into the cytosol in a functional form [29,36,63]. Recently, it has been proposed that a molecular complex comprising the thioredoxin reductase/thioredoxin (Trx/TrxR) system and the chaperone Hsp90, which are both present on the cytosolic side of the SV membrane, fulfills both the reduction and the refolding of the L chain [35,64,65]. Once released in the cytosol, the L chains of the different serotypes cleave distinct peptide bonds of specific SNARE proteins [30,31]: BoNT/B, /D, /F and /G hydrolyze VAMP-1/2, whereas BoNT/A and BoNT/E cleave the membrane protein SNAP-25. BoNT/C was the unique toxin found to cleave more than one SNARE member, that is, both SNAP-25 and syntaxins-1/2 (Figure 2 and Table 1). However, with the recent discovery of novel BoNTs (discussed below), this feature may no longer be considered as a BoNT/C peculiarity. In any case, the cleavage of any one of these proteins blocks the neuroexocytosis process, causing botulism and, in general, the various membrane fusion events mediated by these proteins in eukaryotic cells. The intraneuronal lifetime of the L chains is a primary determinant of the persistence of both BoNTs’ pharmacological activity and of botulism severity and duration. The order of duration of action in mice and humans is: BoNT/A~BoNT/C > BoNT/B~BoNT/D, BoNT/F, and BoNT/G~BoNT/E [1]. However, BoNT/D is almost inactive in humans but very potent in rodents [66].

## 3. Botulinum Neurotoxins Variability and Classification

Emile van Ermengem was the first to isolate a *C. botulinum* strain producing a neuroparalyzing agent in 1895 [67,68]. Five years later, by using antisera generated using neurotoxins from specific *C. botulinum* strains, Leuchs demonstrated that antisera were not cross-neutralizing and that BoNTs could be antigenically different [15]. With the same approach, a total of seven different toxin types were isolated in the following 50 years (in chronological order: BoNT/C, BoNT/D, BoNT/E, BoNT/F and BoNT/G), leading to the definition of BoNT serotypes and to their historical classification. Serotype definition was challenged when an antiserum raised against BoNT/C (strain Stockholm) neutralized BoNT/D (South Africa strain). Later, it was shown that such a cross-reactivity was due to the genetic recombination of bacterial strains, which led to the generation of a mosaic toxin composed by the L chain of BoNT/D and the H chain of BoNT/C [69,70]. Thereafter, a new designation was introduced for the neurotoxin produced by the strain “South Africa” (i.e., BoNT/DC) to indicate the chimeric nature of the toxin. Several mosaics between BoNT/D and BoNT/C with various domain combinations were subsequently characterized [71,72,73,74].

With the development of next generation sequencing, the genetic analysis of clostridial strains responsible for human and animal cases of botulism became systematic. As a result, by sequencing thousands of biological samples, it was observed that neurotoxigenic clostridia have extensive heterogeneity in terms of genome organization, clustering of toxin genes and, most importantly, toxin’s amino acid sequence composition [14,75,76,77,78,79]. Novel toxins have been isolated and termed “subtypes” (dubbed as the parental serotype followed by a number; e.g., BoNT/A1, BoNT/A2, etc.), indicating a BoNT similar to the parental serotype but divergent for amino acid composition. Currently, more than 40 unique BoNTs have been identified, which display heterogeneity ranging from very little (<1%) to higher than 35% [15,76]. Variability represents another Janus-faced property of BoNTs: on one hand, it challenges the current attempts of controlling BoNT pathogenicity via immunological methods; on the other, some of these subtypes could be endowed with peculiar features to be exploited for therapeutic purposes [1,34]. Indeed, apart from antigenicity, variations in the amino acid composition can significantly alter BoNTs’ toxicological features. Even minor amino acid replacements can modify neuronal binding capability/selectivity, catalytic activity, intraneuronal lifetime of the L chain or its translocation efficiency. Despite that functional characterization of these subtypes has been very limited so far, some examples have been put forward. Most of the studies have been focused on BoNT/A toxins due to the large use of BoNT/A1 in human therapy [80,81,82]. BoNT/A2 displays faster entry into neurons and elicits a faster paralysis with lower spreading upon local injection than BoNT/A1, two very appealing features for human therapy [83,84,85]. BoNT/A3 is much less potent and persistent than BoNT/A1, whereas BoNT/A4 displays poor protease activity [81,84]. BoNT/A5 seems to have a toxicological profile similar to BoNT/A1 [84], while BoNT/A8 has reduced catalytic activity and overall lower toxicity [86]. Much less work has been carried out for other subtypes. One relevant result is about the different protease activity of BoNT/F5 compared to BoNT/F1 that, even though cleaving the same substrate VAMP-1/2, hydrolyzes distinct peptide bonds (Figure 2 and Table 1) [87]. Another interesting investigation showed that BoNT/B2 has much higher affinity than BoNT/B1 for synaptotagmin-2 [88]. This is relevant for therapy because BoNT/B2 may have a more favorable pharmacological profile in the treatment of neuromuscular disorders than BoNT/B1, which is currently approved for human therapy but displays some side effects due to the spreading to autonomic nerves [89]. In fact, BoNT/B2 is expected to display a preferential binding to motor nerve terminals, which constitutively express synaptotagmin-2 but not synaptotagmin-1 [90], rather than to autonomic nerve terminals that predominantly express synaptotagmin-1 [91].

Since BoNT variants are being identified with increasing frequency and they can differ to a very minimal extent, the nomenclature around these neurotoxins is becoming difficult to manage. To avoid that, toxins with identical sequences may be given different designations or, conversely, novel toxins with unique sequences may be given the same designations in 2017, an ad hoc committee consisting of over 20 researchers proposed that (i) a centralized procedure should be undertaken to designate putative novel toxins [15] and that (ii) newly discovered neurotoxin variants must differ from other neurotoxins by more than 2.6% at the amino acid level. Yet, this designation does not take into account the biological, functional and toxicological properties of the subtypes which could take place even with sequence variation below this arbitrary threshold. As an example, replacement of only three amino acids in BoNT/C L chain almost completely abrogates SNAP-25 cleavability (without significant alteration of syntaxin cleavability) and this remarkably impacts BoNT/C potency and intraneuronal lifetime [92,93]. Though these mutants are not naturally occurring, their activity strongly suggests that even very minor modifications can significantly affect BoNT toxicological properties. 

## 4. BoNT/H (Also Known as BoNT/FA or BoNT/HA): A Complicated Story

In 2013, after four decades from the isolation of the last BoNT serotype (BoNT/G), a new toxin produced by a bivalent *C. botulinum* strain was isolated from a human infant botulism case. This toxin fulfilled the classic criteria of “new serotype” because it was not neutralized in the mouse bioassay by a mixture of monovalent antitoxins or by the US Army heptavalent antitoxin. Accordingly, it was designated as BoNT/H [94,95]. However, the designation was questioned [96] and a genetic investigation of the toxin gene revealed that BoNT/H shares ≈80% identity with BoNT/F5 in its catalytic domain, ≈84% with BoNT/A1 in its binding domain and displays a translocation domain similar to BoNT/F1 [97]. This suggests that, from a genetic point of view, BoNT/H is a BoNT/F5A1 hybrid, and functional studies—showing that its protease activity is identical to that of BoNT/F5 against VAMP [98] and by binding to SV2 as BoNT/A1 [99]—corroborated this possibility. Moreover, since a second study showed that BoNT/H can be neutralized by existing antisera [97], BoNT/H was renamed “BoNT/FA”. However, neutralization efficiency with respect to reference toxins is much lower, ranging from 20-fold to >500-fold, depending on the antitoxin used. Notably, by using monoclonal antibodies, it was shown that only antibodies specific for BoNT/A, but not for BoNT/F, bind to BoNT/H (BoNT/FA) and display neutralization capacity [100], suggesting that the L-HN of the toxin is immunologically unique. As a consequence, a third name, BoNT/HA, was proposed to underline the absence of BoNT/F immunological properties and the high homology to the BoNT/A binding domain [99]. 

From a functional point of view, BoNT/H (i.e., BoNT/FA or BoNT/HA) is very active on cultured neurons with no species-specificity (human vs. rodents) and its light chain is markedly more efficient than that of other VAMP-cleaving toxins. At the same time, its toxicological profile in vivo is unusual, as it displays a relatively low potency and a slow progression of botulism symptoms [101]. Slow onset of paralysis is also observed when BoNT/H (i.e., BoNT/FA or BoNT/HA) is locally injected, yet it unexpectedly produces a relatively persistent duration of action, even slightly longer than BoNT/B [102]. 

Altogether, these results suggest that BoNT/H (i.e., BoNT/FA or BoNT/HA) is a very peculiar toxin and further demonstrate that BoNT variants can be endowed with unique serological, genetic and functional features which make difficult their classification by arbitrary definitions. In general, the complicated story of BoNT/H (i.e., BoNT/FA or BoNT/HA) perfectly embodies the challenge that researchers have to deal with to classify toxins trying to reconcile immunological, genetic and functional properties. Currently, three designations for BoNT/H, based on one of these features, are indistinguishably used and indistinguishably (not) accepted [15]. Such a nomenclature may be complicated to manage and may become confusing if novel toxins with similar requirements are discovered.

## 5. Identification and Characterization of BoNT/X

In 2017, Zhang et al. found in the chromosome of a *C. botulinum* (strain 111) the gene of a putative neurotoxin displaying significant divergence with respect to known BoNTs. Strain 111 was responsible for a case of infant botulism in Japan, where BoNT/B2, encoded on a plasmid, was identified as the neurotoxic agent [103]. This putative toxin displayed the typical BoNT molecular architecture (Figure 1) and several key features essential for neurotoxicity, including the interchain disulphide bond, the residues forming a ganglioside binding pocket and the metalloprotease consensus sequence (Table 1). Even though it remains to be established whether this putative BoNT is actually expressed by the bacterium (loss of the plasmid encoding for BoNT/B2 abrogates toxicity [104]), a recombinant derivative cannot be recognized and neutralized by existing antisera. Accordingly, if this toxin would be naturally produced it could be certainly considered a new serotype and designated as BoNT/X [105]. Using a recombinant L chain and mass spectrometry, it was found that BoNT/X cleaves VAMP-1/-2/-3 at a unique peptide bond (R66–A67), differently from all other VAMP-specific BoNTs (Figure 2 and Table 1). Unexpectedly, BoNT/X also cleaves VAMP-4 (K87–S88), VAMP-5 (R40–S41) and Ykt6 (K173–S174), three SNARE proteins involved in various membrane-trafficking events among different cellular compartments but not directly involved in neuroexocytosis. VAMP-7, VAMP-8 and Sec22b are instead resistant. Given that the SNARE-selectivity of BoNTs relies on multiple, extended and very specific interactions between the L chain and the substrate [1,106,107,108,109], the ability of BoNT/X to cleave such a copious number of VAMP isoforms is very surprising. Even more surprising is the fact that, notwithstanding such a broad specificity, LC/X chain cleaves VAMP-2 with a 10-fold higher efficiency than LC/B [110]. Moreover, a BoNT/X derivative consisting of two nontoxic fragments enzymatically ligated (LC-HN and HC) can enter neurons and cause flaccid paralysis in vivo, thus suggesting that it can, in principle, cause botulism.

Further studies are needed to determine: (i) Which is (are) the receptor(s) mediating cellular uptake? (ii) What are the functional consequences of the simultaneous inactivation of so many VAMP isoforms in neurons? and (iii) What is the potency in vivo relative to other BoNTs? Moreover, future structural and biochemical analyses may explain in which way BoNT/X light chain can interact and cleave so many different substrates. These data will be helpful both to study the physiological role(s) of specific neuronal VAMP isoforms and to possibly engineer BoNT proteases against specific target(s) for novel applications.

## 6. Discovery and Biological Characterization of the First Non-Clostridial Botulinum-Like Toxin

In 2015, Mansfield et al. found in the genome of *Weissella oryzae* SG25 an open reading frame (then dubbed *Wo-orf1*) sharing substantial nucleotide sequence homology with *bont* genes but lacking some essential features of BoNTs and the additional genes encoding for the neurotoxin accessory proteins typical of BoNT gene clusters [111]. *W. oryzae* is a Gram-positive bacteria member of the phylum Firmicutes, like *Clostridium*, but it belongs to the separate class of Lactobacillales. It was first isolated from fermented food and rice grains, an ecological niche shared with *C. botulinum* [112]. The authors suggested the possibility that *Wo-orf1* was laterally transferred into the *W. oryzae* genome from an unknown source, possibly from a neurotoxigenic *Clostridium* present within the same environment.

Despite low sequence identity (14–16%), structural modeling of Wo-ORF1 predicted substantial folding homology with BoNTs, with the putative L chain (Wo-ORF1-LC), HN domain and the HC domain (Wo-ORF1-HC) folding very similarly to the corresponding domains in BoNT/B. In addition, Wo-ORF1-LC contains the critical HExxH zinc-coordinating motif (Table 1) and the additional Arg and Tyr (residues 369 and 372 of BoNT/B) of the second shell of zinc coordination, essential for the metalloprotease activity [113]. At the same time, significant differences exist. Wo-ORF1 does not display the conserved cysteine subtending the interchain disulphide bond, essential to generate the mature dichain structure typical of BoNTs (Figure 1). Moreover, Wo-ORF1-HC does not contain the key amino acids arranging the GBP and the interface for known protein receptors (synaptotagmin or SV2). Nevertheless, in order to characterize the biological features of Wo-ORF1, Wo-ORF1-LC and Wo-ORF1-HC were produced by recombinant methods [114]. In keeping with structural homology but substantial amino acid divergence, Wo-ORF1-LC and Wo-ORF1-HC were not immunoreactive with standard antisera, except for a very weak cross-reactivity with anti-BoNT/C and -BoNT/D [114]. Importantly, Wo-ORF1-LC was found to cleave VAMP-2 at the peptide bond W89–W90 located within the juxtamembrane segment. This site plays an essential role during neurotransmitter release [115,116] and it is conserved in several VAMP isoforms (VAMP-1/-3/-4/-8) of vertebrates (human, mouse, rat and rabbit), invertebrates (*C. elegans*) and plants. Wo-ORF1-LC activity is thus expected to cause the release of the entire cytosolic domain of VAMP-2, thus preventing the assembly of the SNARE complex and the subsequent process of neuroexocytosis. Building upon these results and on the lack of significant cross-reactivity of Wo-ORF1 subdomains with existing antisera, Zornetta et al. propose that the *orf-1* gene of *W. oryzae* codes for a novel BoNT-like toxin tentatively termed BoNT/Wo [114]. Notably, whether and in which environmental conditions *W. oryzae* naturally expresses BoNT/Wo should be investigated. In fact, given the significant differences in the binding domain, it is more than possible that BoNT/Wo may display, besides distinct binding and internalization mechanisms, a host target different from vertebrate, a possibility also indicated by its peculiar cleavage site. A careful analysis of *W. oryzae* ecology and the generation of a full-length toxin may help to address these issues.

## 7. The First Complete *Bont* Locus in a Non-Clostridial Organism

The finding of the *orf1* gene in *W. oryzae* SG25 suggests that BoNT homologous sequences may be more widespread in the environment than previously appreciated. Indeed, the presence of BoNT-like genes in unusual species indicates that *bont* loci may move into different bacteria via gene transfer events. In line with this possibility, Brunt et al. and Williamsons et al. have recently reported the presence of a complete *bont* gene cluster, tentatively named *eBoNT/J*, in the genome of *Enterococcus* sp. 3G1_DIV0629, isolated in South Carolina (USA) from cow feces [117,118]. The most closely related species of this strain is *Enterococcus faecium*, a commensal bacterium often found in the gut lumen of terrestrial animals (including humans), yet strain 3G1_DIV0629 is the unique out of >1000 *Enterococcus* species in containing the *bont* gene cluster. Interestingly, species of *Enterococcus* like clostridia belong to the phylum of Firmicutes, but to the order of Lactobacillales like *W. oryzae*, indicating that the *bont* genes may have some preferential tropism for this order. The predicted neurotoxin gene product, despite low sequence identity (38% similar to BoNT/X), includes all the functional domains and shares many features of a typical BoNT, including the two cysteines for the interchain disulfide bond, the ganglioside binding pocket and the metallopeptidase motif (Table 1). Conversely, the binding domain, like in BoNT/X and BoNT/Wo, is rather different, indicating a peculiar targeting mechanism. Moreover, all open reading frames of the toxin cluster are intact, suggesting a likely possible expression.

Back-to-back with the publication of Brunt et al., Zhang et al. published a paper reporting the biological characterization of the same putative neurotoxin (eBoNT/J), that was now dubbed “BoNT/En” [119]. They found that BoNT/En (i.e., eBoNT/J) is not recognized by the standard BoNT antisera (including BoNT/X) and that it cleaves in vitro VAMP-1/-2/-3 to a conserved peptide bond A67–D68, very close to BoNT/X cleavage site. Interestingly, Zhang et al. showed that BoNT/En (i.e., eBoNT/J) also cleaves, although less efficiently, syntaxin-1B and syntaxin-4 (K191–D192) and SNAP-23/25 (K69–D70) (Table 1). Syntaxin cleavage is not maintained in living neurons, where only VAMP-2 and SNAP-25 are hydrolyzed. This result indicates BoNT/En (i.e., eBoNT/J) as the first toxin that potentially cleaves all three SNARE proteins. In addition, it is the first BoNT reported to cleave SNAP-25 at the N-terminus rather than at the C-terminus. BoNT/En (i.e., eBoNT/J) seems not to be toxic in mice, however, a chimeric toxin composed by the H chain of BoNT/A and the L chain of BoNT/En leads to paralysis and induces botulism symptoms, suggesting that this putative BoNT may display a different binding mechanism or even a different cell target. 

The discovery of a botulinum-like toxin in *Enterococcus* species may be more than the tip of a large iceberg, with the possibility that many others will be identified in the future.

## 8. Engineering BoNTs: Potential for Novel Therapeutic Applications

Since the pioneering application in ophthalmology by Scott et al. in 1990 [120,121], the clinical use of BoNTs has been continuously evolving. In modern medicine, BoNTs represent multipurpose therapeutic agents for the treatment of neurogenic movement disorders, neurosecretory, urologic, orthopedic, gastrointestinal, dental and pain conditions, with a large part of these applications being off-label [1,9]. Moreover, BoNTs have become very popular in cosmetics. Notably, all these applications are almost exclusively based on the use of BoNT/A1, as it provides the most persistent activity. Only in very few cases, almost exclusively due to the development of resistance to BoNT/A1, BoNT/B1 is used but with less success because it displays a much less convenient pharmacological profile, at least for neuromuscular disorders, as it requires higher doses and it has a much shorter duration [1,89]. Eleopra et al. were the first to explore the potential use of other BoNTs for human therapy [122,123,124,125]. It was shown that BoNT/C has a pharmacological profile similar to BoNT/A1, with similar potency and equal duration of action, whereas BoNT/E1 and BoNT/F1 produce a much shorter effect, even shorter than BoNT/B1. Intriguingly, BoNT/D, which is the most potent toxin type in rodents [1], was reported to be almost completely ineffective at the human neuromuscular junction and to cleave human VAMP-1 to a very low extent, explaining why very few cases of human botulism were linked to this toxin [66,126,127,128,129]. A unique study showed comparable activity of BoNT/D to BoNT/A, BoNT/B and BoNT/E [130]. Since insensitivity to BoNT/D by human muscles has been linked to a point mutation in VAMP-1, the study of Anderson opens up the possibility that polymorphism on human VAMP-1 gene may be responsible for this discrepancy. However, a recent study reported that human SNARE proteins do not present polymorphisms and are rather refractory to mutations, thus ruling out this possibility [131]. Altogether, these results indicate that BoNT/C represents a suitable alternative to BoNT/A, and that other BoNTs, like BoNT/E or /F, may be used when a short duration of action is preferable. BoNT subtypes are also considered an interesting avenue to explore. Indeed, among the many variants isolated so far, only a few have been characterized (mainly of the BoNT/A family) and they were found to display significantly different toxicological (thus pharmacological) properties [81,82,83,84]. In general, subtypes represent a potential goldmine to improve the current clinical use of BoNTs.

An important aspect of BoNTs is their protein nature and, in general, the possibility to be easily engineered and produced by recombinant methods [132]. Several laboratories are working in this direction and reported interesting results. Chen et al. and Sikorra et al. performed mutagenesis on BoNT/E and BoNT/A, respectively, to adapt their L chains to human SNAP-23, normally refractory to cleavage. They isolated cleaving mutants representing a solid base for the development of novel derivatives for the treatment SNAP-23-mediated hypersecretory disorders [133,134]. Wang et al. generated BoNT/AE chimeras (BoNT/A light chain, BoNT/E binding domain) to prolong the activity of BoNT/E [135]. BoNT/AB chimeras (BoNT/A light chain, BoNT/B binding domain) were used by Wang et al. to reduce exocytosis from non-neuronal cells expressing the BoNT/A-acceptor and utilizing VAMP, but not SNAP-25, in exocytosis [136], and by Kutschenko et al. to combine the high binding affinity of BoNT/B for autonomic nerve terminals and the long-lasting activity of BoNT/A light chain, thus developing a toxin to improve the treatment of autonomic disorders [137]. Pirazzini et al. produced a BoNT/B1 mutated in the translocation domain, which displayed higher potency than the wild type [138]. Tao et al. engineered the binding domain of BoNT/B1 to increase its affinity for human synaptotagmin-2, obtaining a mutant toxin with much higher activity in neurons expressing human synaptotagmin II [139]. More recently, Zanetti et al. showed that BoNT/C mutants with decreased activity against SNAP-25 display markedly low lethality but still maintain a long lasting neuromodulatory activity at the neuromuscular junction [93]. Accordingly, syntaxin-specific BoNTs represent a putative class of neurotoxins that can attenuate nerve terminal activity without affecting the overall functionality of the muscle, a picture of remarkable value in BoNT therapy.

Furthermore, the multidomain architecture makes BoNTs suitable platforms to be exploited for intracellular delivery of exogenous proteins. Proof of principle was provided by Bade et al., who engineered BoNT/D, fusing various proteins to its L chain. Translocation of protein cargoes inside neuronal cytosol was efficient, with the only constraint that the cargo must be able to undergo unfolding [140]. Similar examples have been also provided by others [141,142,143,144]. Alternatively, specific fragments of BoNTs can be produced and rejoined by using a “protein-stapling” technology to combine distinct parts of different BoNTs (and possibly other targeting proteins), still maintaining enzymatic activity of the L chain [145,146].

In general, these studies show how a few substitutions in amino acid sequence can functionally affect BoNTs biological activities, and how versatile BoNTs are to generate novel toxins with new and improved pharmacological features. In this direction, future structural and biochemical data on both the newly discovered serotype BoNT/X as well as on botulinum-like toxins, such as BoNT/Wo and BoNT/En, may open exciting avenues for original and currently unexpected therapeutic applications for human diseases.

## 9. Conclusions

The improvement in DNA sequencing techniques, together with bioinformatics and data-mining tools, are expanding our understanding on the diversity of BoNTs [17]. Surprisingly, BoNTs and BoNT-related genes have been reported in many different bacterial species outside of the genus *Clostridium*, including *Weissella oryzae*, *Enterococcus faecium*, *Mycobacterium chelonae* and *Actinobacteria* [18,111,117,119,147,148]. Despite this breakthrough, most of these toxin genes remain sequenced only and their products have not yet been tested. However, these species may have the potential to produce “BoNT-like toxins” displaying some similarity with the multidomain organization of BoNTs but different specificity, working principles and toxicological features. For instance, a recent report found another putative BoNT-like toxin in the genome of *Chryseobacterium piperi* (designed as Cp1 toxin), which retains structural homology to BoNTs, but it was shown to cause necrotic cell death in a human kidney embryonic cell line, without cleaving any SNARE proteins [148].

In light of these recent findings, it is tempting to speculate that BoNT-like genes and toxins may represent an early branching of BoNT evolution and that the modular domain architecture along with the neurotropism may have emerged more recently, possibly together with the evolution of the vertebrate nervous system. The specificity of BoNTs for neuronal SNARE proteins, dictated by multiple interactions between the L chains and their substrates, may derive from a BoNT ancestor with a much broader protease activity. The broad activity against multiple isoforms of VAMP and several SNARE proteins of the BoNT/X and BoNT/En is in agreement with this possibility. In this scenario, the identification of the substrate(s) of BoNT-like toxins, together with the characterization of their putative cell host(s), may provide new insights about how BoNTs have evolved. At the same time, it cannot be excluded that “classical BoNTs” can have alternative substrates different from SNARE proteins, and their discovery may further contribute to decipher BoNT evolution. However, the most important insight would be that of understanding the biological/ecological role(s) and the adaptive significance of BoNTs in clostridia. Even though it cannot be ruled out that toxigenicity and pathogenicity of BoNTs may be accidental, indeed it is presently unknown what adaptive advantages BoNTs provide to the clostridia life cycle [16]. The predominant explanation relies on the killing of vertebrate animals that are transformed into large anaerobic fermenters, expanding the anaerobic environment where toxigenic and non-toxigenic clostridia can proliferate. Yet, this does not explain the coevolution of so many BoNT serotypes and variants: how and why BoNTs have evolved remains a fascinating issue to be addressed. Future studies may shed light on the biological and toxicological features of BoNTs and BoNT-like proteins, and a comprehensive phylogeny may provide hints on the role of BoNTs and their evolutionary origin.

## Figures and Tables

**Table 1:** Binding receptors, amino acids of the metalloprotease active sites, substrate specificity and cleaved peptide bonds of BoNT variants.

Serotype	Protein Receptor	Ganglioside Receptor	HC-C GBP (SXWY)	Metalloprotease Motif (HExxH)	Substrates	Cleavage Sites
BoNT/A	*N*-glycosylated SV2A-B-C	GT1b GD1a	SNWY	HELIH	SNAP-23	T202–R203
					SNAP-25	Q197–R198
BoNT/B	Synaptotamin-1/2	GT1b GD1a	SKWY	HELIH	VAMP-1	Q78–F79
					VAMP-2	Q76–F77
					VAMP-3	Q63–F64
BoNT/C	none *	GT1b GD1b	(W)KNY	HELNH	SNAP-25	R198–A199
					Syntaxin-1A,-2,-3	K253–A254
					Syntaxin-1B	K252–A253
BoNT/D	*N*-glycosylated SV2A-B-C	GT1b GD1b GD2	(W)VNY	HELTH	VAMP-1	K61–L62
					VAMP-2	K59–L60
					VAMP-3	K46–L47
BoNT/DC	Synaptotamin-1/2	Sialic acid residue	SNYIS	HELTH	VAMP-1	K61–L62
					VAMP-2	K59–L60
					VAMP-3	K46–L47
BoNT/E	*N*-glycosylated SV2A-B	GT1b GD1a	STWY	HELIH	SNAP-23 SNAP-25	K185–I186 R180–I181
BoNT/F	*N*-glycosylated SV2A-B-C	GT1b GD1a	SSWY	HELIH	VAMP-1	Q60–K61
					VAMP-2	Q58-K59
					VAMP-3	Q45–K46
BoNT/F5	Unknown (similar to BoNT/F?)	Unknown (similar to BoNT/F?)	SSWY	HELIH	VAMP-1	L56–E57
					VAMP-2	L54–E55
					VAMP-3	L41–E42
BoNT/H (BoNT/HA, BoNT/FA)	*N*-glycosylated SV2C	Unknown (similar to BoNT/A?)	SNWY	HELIH	VAMP-1	L56–E57
					VAMP-2	L54–E55
					VAMP-3	L41–E42
BoNT/G1	Synaptotamin-1/2	GT1b GD1a	SQWY	HELIH	VAMP-1	A83–A84
					VAMP-2	A81–A82
					VAMP-3	A68–A69
BoNT/X	Unknown	Unknown	SAWY	HELVH	VAMP-1	R68–A69
					VAMP-2	R66–A67
					VAMP-3	R53–A54
					VAMP-4	K86–S87
					VAMP-5	R40–S41
					Ykt6	K173–S174
BoNT/Wo	Unknown	Unknown	Not present **	HEMTH	VAMP-2	W89–W90
BoNT/En (eBoNT/J)	Unknown	Unknown	SAWY	HELCH	SNAP-25	K69–D70
					VAMP-1	A69–D70
					VAMP-2	A67–D68
					VAMP-3	A54–D55
					Syntaxin-1B	K145–D146
					Syntaxin-4	K191–D192

* BoNT/C binding and internalization may be independent from protein receptors. Polysialogangliosides involved in toxin internalization are also indicated. BoNT/DC is unique among BoNTs, not necessarily needing the interaction with polysialogangliosides. BoNT/X and BoNT/En contain the ganglioside binding pocket domain (GBP), but the dual receptor binding process has not been yet proven. ** Conserved residues forming the ganglioside binding pocket (GBP) are not present in the putative binding domain of BoNT/Wo, which may have a different binding mechanism. The enzymatic substrates and cleavage sites experimentally proven are indicated with mouse numbering. SNAP: synaptosomal-associated protein; VAMP: vesicle-associated membrane protein.
